# Pesticides use and health impacts on farmers in Thailand, Vietnam, and Lao PDR: Protocol for a survey of knowledge, behaviours and blood acetyl cholinesterase concentrations

**DOI:** 10.1371/journal.pone.0258134

**Published:** 2021-09-30

**Authors:** David Hughes, Wisit Thongkum, Kukiat Tudpor, Niruwan Turnbull, Nachalida Yukalang, Vanphanom Sychareun, Thang Van Vo, Latt Latt Win, Alan Watkins, Sue Jordan

**Affiliations:** 1 Faculty of Medicine, Health & Life Science, Swansea University, Swansea, United Kingdom; 2 Faculty of Public Health, Mahasarakham University, Tambon Khamriang, Amphur Kantarawichai, Mahasarakham, Thailand; 3 Faculty of Public Health, University of Health Sciences, Vientiane, Lao PDR; 4 Institute for Community Health Research, University of Medicine and Pharmacy, Hue University, Hue City, Thua Thien Hue Province, Vietnam; 5 Department of Pharmacology, University of Medicine, Taunggyi, Myanmar; Duy Tan University, VIET NAM

## Abstract

Pesticides use in Southeast Asia has increased steadily, driven by the growth of large-scale commercial farming, as well as a desire to maximise food production in rural subsistence economies. Given that use of chemical pesticides, such as organophosphates and carbamates, has known potential health impacts, there are concerns about the safety of agricultural workers, and a need for a better evidence base to underpin regulation and worker education. This study, undertaken in 9 districts in Lao PDR, Thailand and Vietnam, will interview agricultural workers to investigate how they use pesticides, their knowledge of risks and self-protective practices, and their self-reported illness symptoms. In each district researchers will recruit and interview 120 participants engaged in vegetable farming, who have recently used pesticides, making a total of 1080 subjects divided equally between the three study countries. Workers’ degree of pesticides exposure will be determined from acetyl cholinesterase concentrations in capillary blood samples collected using field test kits, and these data will be analysed together with the interview findings. Country findings will be compared and contrasted, and general patterns noted. Knowledge gained about risky behaviours, self-protective practices and degree of association with serious pesticides exposure will assist policy makers and inform health improvement programmes.

## Introduction

### Genesis of the project

The planned study is a comparative investigation of health impacts of pesticides use to be completed by teams from universities in Thailand, Lao PDR and Vietnam, with support from Swansea University, UK. The idea for a study of this type came from Mahasarakham University (MSU) Thailand, based on earlier work by Kukiat Tudpor and associates [1]. A plan to include Myanmar had to be abandoned due to major internal disorder associated with the 2020 military coup, but the Myanmar research lead remains in our network team and has contributed to background research on pesticides policy and agricultural practices in Southeast Asia. ‘Seed corn’ funding for a comparative study was provided by MSU, but the schedule and scope of work was disrupted by the COVID-19 pandemic. Swansea University had been involved in early project planning, and was eventually successful in securing a UK Global Challenges Research Fund (GCRF) award to allow the project to progress. During the period covered by the original project plan, only MSU was able to conduct fieldwork, and that research is now being treated as both a feasibility and a pilot study, which will assist in the full implementation of the research plan set out below. This pilot work has been helpful in focusing work on agricultural workers, rather than continuing with the original plan to also include testing of fruit and vegetable samples sent to market, and in refining the structured interview instrument.

### Background

Pesticides are used extensively in agriculture around the world to improve crop yields by controlling insects, fungi, molluscs, and rodents [2]. Their use is especially prevalent in farming regions in Asian countries, as grains, vegetables and fruit are the main products that support families in rural subsistence economies. Chemical pest control has been utilized for centuries to increase and protect fruit and vegetable crops, and there may be resistance to changing what are seen as long-established practices [3–5]. There is also a powerful economic incentive for farmers to increase production of food as a way of increasing incomes. However, in the longer term, chemical pesticides have been shown to have harmful effects on the ecological system and there is mounting evidence of damage to human health [6–12]. Pesticides can contaminate rivers, lakes and oceans, which in turn may well pollute the natural environment and food chain. Potential risks to human health documented in the literature include: acute neurologic poisoning, chronic neurodevelopmental impairment, cancer, reproductive dysfunction, and possibly dysfunction of the immune and endocrine systems [7]. Pesticide residues on agricultural products can be transferred directly to humans, with deleterious health effects [13,14]. Moreover, both soil contamination and water contamination may also have negative health effects [15,16]. Apart from effects on health through diet, those who handle and apply pesticides may be affected, as absorption occurs through the skin, lungs and mucous membranes. Farm workers, including sprayers and mixers, are a high-risk group whose health may be seriously compromised [17].

All three countries in the study are major food producers and exporters. Large-scale commercial farming has grown in Asia in recent decades [18–20], with major impacts on subsistence farmers and local communities; this has led to a dramatic increase in pesticides use. All the countries have been affected by recent social and economic disturbances. For example, the Thai government’s attempts to guarantee rice prices for farmers led to major political turmoil, and support for small farmers remains a challenging development issue. It has been estimated that the volume of pesticides used in SE Asian countries increased by approximately 400 per cent in the first decade of the new millennium [3]. Thailand is reported to be the highest user of imported pesticides by volume in the region [21], while Vietnam has the highest application rate of 16.5kg/hectare [22]. However, analysis by these latter authors shows that the acceleration in the use of pesticides has been higher in recent years in Cambodia and Lao PDR than in Vietnam and Thailand. This is largely because, before 2000, the volumes used (kg per hectare) had remained low in Lao PDR (close to zero) and Cambodia, but had already risen in Thailand and Vietnam. Consequently, in the decade to 2012, and based on a calculation of exponential growth rates, pesticides use increased by 61% each year in Cambodia, 56% in Lao PDR, 9.8% in Vietnam and 6.8% in Thailand. Throughout this decade, each country had a different pattern of change: the use of pesticides remained low in Lao PDR, rose in most (but not all) years in Thailand, accelerated from ~2008 to 2012 in Cambodia, and fluctuated in Vietnam, peaking in 2008 and 2012 [22].

A follow-on study by the same research team found that 100% of sampled vegetable farmers in Vietnam, 73% in Cambodia and 59% in Lao PDR were ‘overusing’ pesticides in the sense of applying levels in excess of a profit-maximizing optimum [23]. It has been reported that many producers purchase pesticides without proper training regarding their application [24]. This has prompted widespread public concern about possible harm to health.

Most pesticides are synthetically-produced organic and inorganic chemical compounds, although biological agents are also coming into wider use [25]. The most commonly imported pesticides are organophosphates (OPPs) and carbamates [26]. OPPs and carbamates kill insects by blocking acetylcholinesterase (AChE), an enzyme that catalyses hydrolysis of a neurotransmitter acetylcholine, resulting in overstimulation of the neuromuscular system and the parasympathetic nervous system. Exposure to OPPs by inhalation or ingestion with or without food is toxic to the human body. It can be detected by a reduced AChE concentration in capillary blood. Acute OPP poisoning symptoms include: increased salivation, diarrhoea, vomiting, muscle tremors, gastrointestinal upset, and confusion [27]. The onset of symptoms can start within minutes or hours and last for days to weeks. In the long term, lower-dose exposure to OPPs has been reported to cause polyneuropathy and cardiovascular diseases [28]. Carbamates can also pose both health and environmental hazard, including through groundwater contamination [29] and food [29,30].

The health impact of pesticide use has been a topic of heated debate for many years in Asian countries, both at national and local government levels. There is a need to prevent adverse health effects as part of a wider pesticides management strategy, and there would be major advantages in establishing a framework for cooperation within the Association of Southeast Asian Nations (ASEAN). At present, countries in the Mekong region have only their differing national strategies, and it would be valuable to compare approaches and develop a more uniform approach that takes the best ideas from each country.

Although all three countries in the study have developed legal frameworks to regulate pesticides use, limitations on what is permissible and enforcement are generally not strict. In 2009, the Food and Agriculture Organisation of the United Nations launched a project to encourage ‘harmonisation’ of pesticides regulation in Southeast Asia [31], in which all three study countries participated and produced work plans. Currently all are signatories to the Basel (1989), Rotterdam (1998) and Stockholm (2001) Conventions, which control aspects of the transboundary movement, disposal, trade and use of pesticides. Myanmar which we hope to include in future studies, has not so far ratified the Rotterdam Convention. All continue to permit the use of organophosphates and carbamates on crops, but have introduced legislation on registration of products, licensing, import controls, and labelling requirements [see 32]. All rely on the relevant ministries to oversee pesticides use, and none have an independent agency to ensure public safety such as the United States’ Environmental Protection Agency [33]. According to a recent joint UN FAO and WHO report, there is an urgent need for improved evaluation and risk assessment to regulate pesticide residue levels in food [34].

Developing an effective policy response to negative impacts of pesticides use is a challenging task, with no single approach fitting all contexts [35]. Thailand has probably moved further than Vietnam or Lao PDR to develop a national strategy, while Vietnam has introduced an innovative pesticide tax, and there are promising training schemes for pesticide retailers in Thailand and Vietnam [22]. Thailand’s 2018–2037 strategy has identified increasing the quality and quantity of agriculture products as one of five key development targets. The strategy proposes five key principles to safeguard food quality and avoid harm: 1) farming that reflects local identity; 2) safe farming; 3) biological farming; 4) regulation of processed agricultural products; and 5) smart farming [36]. This strategy puts safety at the centre of its plan for improving agriculture products. It would transform agricultural practices so that farmers adopt animal husbandry and crop cultivation methods that do not damage the environment or human health. Regarding pesticides, sustainable agriculture requires an effective pesticide management system that follows international standards. Such a system, if developed, could provide a model for neighbouring countries and help introduce a more consistent approach to pesticide control and surveillance in ASEAN countries.

A scoping exercise for the present project found many single-country studies of agricultural workers’ pesticides use in Mekong region countries, but a paucity of comparative studies.

Interview studies that examine some combination of farm workers’ agricultural methods, pesticides and health knowledge, attitudes, health protective practices, and self-reported illnesses have been undertaken in Thailand [37–40], Vietnam [41–44], Lao PDR [45,46], Myanmar [47–49], and Cambodia [50]. A small subset of studies use field capillary blood tests to measure farm workers’ AChE levels, thus adding objective data on degree of pesticides exposure to interview findings [1,51–55]. Taken together, the single-country studies give a reasonable picture of farming practices and patterns of pesticides use across the region, but because of variations in data collection, study sample size, farm types and crops covered, and degree of rigour, further research is needed for comparative analysis.

Comparative studies that use a standardised methodology to compare the situation in more than one Mekong region country are thin on the ground. The most comprehensive study found in our preliminary review is Schreinemachers and colleagues’ 2017 research on small-scale vegetable farmers in Vietnam, Lao PDR and Cambodia [56]. The researchers utilised a researcher-administered questionnaire to gather data from 900 households in 93 villages. Questions covered knowledge, attitudes and practices (KAP) related to pesticides use as well as self-reported symptoms of pesticides poisoning. As mentioned earlier, a team with the same lead researcher has also investigated volumes of pesticides used in Vietnam, Lao PDR and Cambodia utilising a later survey of 1000 households in 93 villages in the three countries. [23]. The present study fills a gap by providing current comparative data on a different three country set of Mekong region countries. It concentrates more on health impacts, including attention to both self-reported symptoms and examination of capillary blood samples for pesticides exposure. We believe this triangulated design, based on both self-reported health status and objective blood test data, will reduce the possibility of common method bias (CMB) by using different procedures to measure dependent and independent variables [57].

## Aim and objectives

The general aim of the study is to investigate the potential health impacts of pesticides use on agricultural workers.

The specific objectives are:

1. To estimate the prevalence of high OPP and carbamate exposure via AChE concentrations found in agricultural workers’ capillary blood samples.
2. To determine the proportion of workers with suboptimal behaviours that predict high exposure to pesticides.
3. To examine workers’ health knowledge regarding pesticides-related risks, the prevalence of heath-protective behaviours, and self-reported ill health.
4. To investigate possible associations between agricultural practices, health knowledge, health-protective practices and reports of ill-health, and the concentration of cholinesterase in capillary blood samples.

## Materials and method

### Overview of survey and blood cholinesterase assays

Our study investigates agricultural workers’ self-reported use of pesticides, health knowledge, health protective behaviours, and health status, in combination with the measurement of pesticide exposure in capillary blood samples. Direct questioning of workers is reported to be the most common research method for assessing pesticide exposure [58], as was confirmed in our own scoping exercise [1]. In our study pesticide exposure will be assessed via measured cholinesterase concentrations using commercial field test kits (see below). Empirical fieldwork will be completed in selected localities in Thailand, Vietnam, and Lao PDR, so as to facilitate comparison of the situation in the three countries.

Researchers in each country will purposively select three suitable districts within the province in which their university is located, so that fieldwork will be conducted across 9 such districts (groups of ‘villages’ or ‘local areas’), with a target of 120 interviews per district. A researcher-administered structured interview will be used to collect data on agricultural production, health knowledge, health protective practices and behaviours, and illness episodes. Pesticides exposure will be measured as AChE concentrations in agricultural workers’ capillary blood using field test kits (described below).

### Structured interviews

A researcher-administered structured interview will be used to collect data concerning the nature of agricultural work, pesticides use, workers’ health knowledge, health protective practices, and self-reported experiences of ill health. Interviews of this type are sometimes described as survey interviews or standard interviews, and contain mainly closed-end questions that resemble a questionnaire, but are typically read to the respondent and completed by the researcher. Research assistants (paid research staff or postgraduate students) will be trained to explain the meaning of the various items to workers and complete the form in their presence. The structured interview is adapted from the instrument used in a study by Tudpor and associates [1]. Content validity of items in that instrument were confirmed by an index of conjugate (IOC) calculation. All items had IOC scores greater than 0.5 and an internal reliability coefficient (Cronbach’s α) of more than 0.7. The original Thai interview containing 59 items has been expanded to 83 items in the instrument prepared for the present study. This was modified *inter alia* to use terms and units that apply to all ASEAN countries, to ensure that multi-choice items covered all logical possibilities, and to add additional questions regarding specific pesticides types (insecticides, herbicides and fungicides), crop types, planting periods, self-medication and medicines prescribed by healthcare professionals.

#### Measuring blood cholinesterase concentrations in agricultural workers

Exposure to pesticides can be detected by low acetyl cholinesterase enzyme (AChE) concentrations in capillary blood samples. To measure AChE, we will utilise field kits containing lancets and reactive paper test strips (AChE field test kits, lot U620164, manufactured by the Government Pharmaceutical Organisation [GPO] Thailand). Blood samples (0.2 mL/capillary) will be obtained from a lancet-punctured fingertip of participants, undertaken by volunteer assistants drawn from public health officers, nurses, paramedics, or doctors in the local area. The samples will be allowed to precipitate for 30–45 minutes, followed by the reactive paper test. After 7 minutes, changes in colour on a paper test strip will be recorded using the four categories specified on the standard instruction sheet provided by the GPO with the test kits. The categories are: normal (AChE units per ml >100); safe (87.5–99.9); at risk (75.0–87.4); and probable health impact (<75). The outcome of primary interest is probable health impact.

The blood test results will be analysed alongside the interview data to determine whether participants with AChE levels that indicate probable health impact differ in their agricultural practices, health knowledge, health-protective practices and extent of self-reported illness. We will use descriptive and inferential statistics to explore associations (if any) between key variables, in line with specialist advice on appropriate multivariate analyses.

### Survey design details

#### Sample size basis

The outcome variable for sample size considerations reflects our first objective, to determine the proportion of workers with abnormal concentrations of acetyl cholinesterase (AChE) in capillary blood samples. Recent studies of agricultural workers in Vietnam [59] and Lao PDR [60] show that about 35% of participants had high exposure to pesticides with likely health impacts, as indicated by AChE levels in capillary blood samples.

#### Sampling frame

Background inquiries in Thailand suggest that numbers of households engaged in vegetable farming in districts (villages) in the study province growing these crops for the commercial market do not exceed 200 per district. Our resources will allow us to interview 120 individuals per district (representing about 60% of households in that district), and hence obtain 360 responses from 3 districts in each country.

#### Clustering

We will interview only one person from any one household to avoid data replications on household-level behaviours, such as laundry; this also allows us to use a single intra-cluster correlation coefficient (ICC) for responses from the same district. With no ICC available from our single-district Thai pilot study, we have used the unadjusted median ICC observed in primary care studies across a range of variables: (interquartile range [IQR] 0 to 0.032) [61], and set the ICC here to 0.01. With m = 120 interviews per district, our design effect, calculated as 1+(m-1)*ICC [62] is 2.19; this compares with design effects values ~1.5, as reported in surveys of developing countries [63]. Our confidence intervals are thus inflated by a multiplicative factor of 1.48 over those based on the assumption of independent responses; this adjustment accounts only for clustering within districts (groups of villages).

#### Precision

We are testing the prevalence of cholinesterase concentrations likely to impact on health, estimated at 35% [59,60]. We are able to recruit 360 respondents in each country. We calculate that 360 respondents will allow us to verify this 35% prevalence, with 95% confidence intervals lying between 28% and 42%.

#### Reporting

The data for each country will be reported separately to inform national stakeholders and policy makers. If the data are sufficiently similar across countries, we shall consider a combined analysis in a multilevel model. Otherwise, we shall undertake a meta-analysis to accommodate high heterogeneity.

We believe that abnormal AChE levels anywhere within our estimated range (28%-42%) will suffice as triggers to modify behaviour. Our models will identify behaviours that might usefully be targeted.

### Operational considerations

Certain financial and logistical constraints mean that we cannot apply probability sampling to all levels of participant selection in a way that would have been desirable. The small project budget dictated the decision that each ASEAN country team would limit fieldwork to the province in which the university is located, rather than attempting a country-wide sample. It was further decided to opt for purposive rather than probability sampling of three districts in each province because of exceptional constraints applying at the time of fieldwork. Fieldwork must start in mid-2021 because of deadlines imposed by the funder. It must take place against the background of the COVID-19 pandemic. For these reasons it is necessary to select districts with a sufficient volume of vegetable production in which fieldwork is feasible when travel and safety are considered. We will use already published data to provide information on where the selected districts fit within the spectrum of districts in the study provinces. Because of serious civil disturbances and armed conflict in Shan State we have been forced to abandon the planned Myanmar arm of the study.

### Research ethics

It was a condition of GCRF funding that the project received a favourable ethical opinion from the Swansea University Research Ethics & Governance Committee (Chair Dr Angela Smith; Email A.M.Smith@swansea.ac.uk; website: https://www.swansea.ac.uk/medicine/research/ethics/#bbq=on). The research protocol was approved on 6^th^ January 2021 by the College of Human & Health Sciences Research Ethics Sub-Committee (approval reference number 151220b). The Swansea REC approval allowed project preparatory work to continue, but required additional approval from ethics committees in each of the participating Asian countries before fieldwork in that country could begin. Favourable opinions have been obtained from Mahasarakham University Ethics Committee for Research Involving Human Subjects (Ref. number 127/2020; date 22/4/2020), the University of Health Sciences Research Ethics Committee, Vientiane (Ref. number 137; date 29/3/2021)., and the Institutional Ethics Committee of Hue University of Medicine and Pharmacy (Ref. number H2021/420; date 12/8/2021).

All participants in the study will be given an information sheet and sign a consent form or give a thumb print. The information sheet explains that participation is voluntary and that participants can withdraw at any time, up until a final confirmation of consent after interview and blood tests are completed. It explains the (low) risk of infection associated with finger prick blood tests, offers assurances that confidentiality and anonymity will be preserved, that data will be stored securely, that no blood samples will be retained by the researchers, and that names and addresses will be used only for the purpose of contacting participants to advise of blood test results and, if necessary, to refer them to a local health professional for advice and assistance.

Consent for this study involves consenting to receive blood test results, and agreeing that, if the results show high pesticides exposure, participants’ names can be passed to the local health service. The latter will be done by contacting local staff in the public healthcare systems of the three countries involved. Following the interviews, a health information sheet will be given to participants with advice for handling, using, storing and disposing of pesticides and containers, as above. This will be given to all who complete an interview, regardless of whether they also complete the blood test and are included in the study. As is common in field studies a small reward for respondents will only be payable on completion of the interview and blood test; this is made clear in the information sheet.

### Service user involvement

Health effects of pesticides exposure are a major concern in farming communities, and feedback from those involved in the Thai pilot study helped shape the final research design. The study will enlist the help of local health and social care staff and village guides to recruit and meet participants. There will be feedback and assistance for participants found to have high exposure.

## Fieldwork process

Sampling of cases is based on purposive sampling of three groups of villages/districts in each of the three countries and subsequent recruitment of agricultural workers. The three districts (or groups of villages) will be selected so that taken together they cover a range of small and large farms and spread of vegetable crop types.

Respondents will be sampled randomly from the population of farm workers in the village/district.

1. As a first step health or local government personnel familiar with the district will compile a list of workers involved in vegetable production in the village/district. The list will include all members of households aged 18 and above who work regularly as farmers. Candidates may be natives or migrant workers, but must be fluent in the main language of the study country. They will be encouraged to participate through the offer of a small reward, a cash payment of less than £1 GBP payable on completion of the study. The list will comprise information on name, large or small farm and gender. The number of households and people on the list will be noted.
2. The research team will use a randomising procedure to select from the list 120 first choice workers’ households and 30 reserves (with the latter listed in order).
3. The health or local government staff assisting with the project will be advised of the selection. They will approach the first 120 named candidate households selected by the research team as first choices for inclusion to ask whether a volunteer adult household member is willing to meet with the research team to discuss participation in a research project on pesticides use. Information sheets will be distributed.
4. Those volunteering will be contacted by researchers. The content of the information sheet will be explained and opportunities to ask questions provided. Those indicating their consent to participate will be screened to determine whether they meet the study inclusion criteria (which include a check that only one member per household takes part). Those selected will sign or provide a thumb print on a consent form.
5. The main interview will be conducted either immediately or within a few days
6. When interviews are completed, consent to take blood samples will be confirmed. Only participants who agree to both interview and blood sample will be included in the study. Capillary blood samples will be taken using finger-prick lancets. In Thailand local public health staff measure AChE levels of farm workers routinely as part of a government health promotion initiative, using test kits identical to those purchased from the Thai GPO for the study. If tests were completed within the past 4 weeks these results will be used for this study, as was the case in the pilot study. If no such data are available for consenting participants from the local health service, finger-prick blood tests will be undertaken after the interviews.
7. In the event that candidates from the original 120 selections fail to meet the research team, are found to be ineligible or do not consent to participate in all stages of the study, additional candidates from the reserve list will be approached one by one to make up the numbers.
8. Participants completing the interviews will be given a health protection information sheet detailing dos and don’ts of safe pesticides use.
9. Participants completing the blood tests will be advised of where to seek help if any adverse effects (such as infection) arise. They will also be told how they will be notified of the blood test result and that local health staff will advise them on necessary treatment or behavioural change. They will be given the small study reward.

Agricultural workers eligible for inclusion in the study must:

1. be involved in cultivating vegetables as a main crop for commercial sale for at least 10 months a year (fruit may be included as a secondary crop);
2. have applied pesticides to crops in the last 3 weeks.
3. be aged 18 or above

Workers will be excluded if they:

1. work mainly with organic vegetables or fruits;
2. cultivate hydroponics;
3. cannot communicate in the primary native language of the study country;
4. are from the same household as an already-recruited participant.

We have added the requirement that workers should have used pesticides in the last three weeks because it is known that, following exposure to organophosphates, AChE levels begin to rise to near-normal levels after that period [64].

**Fig 1:**
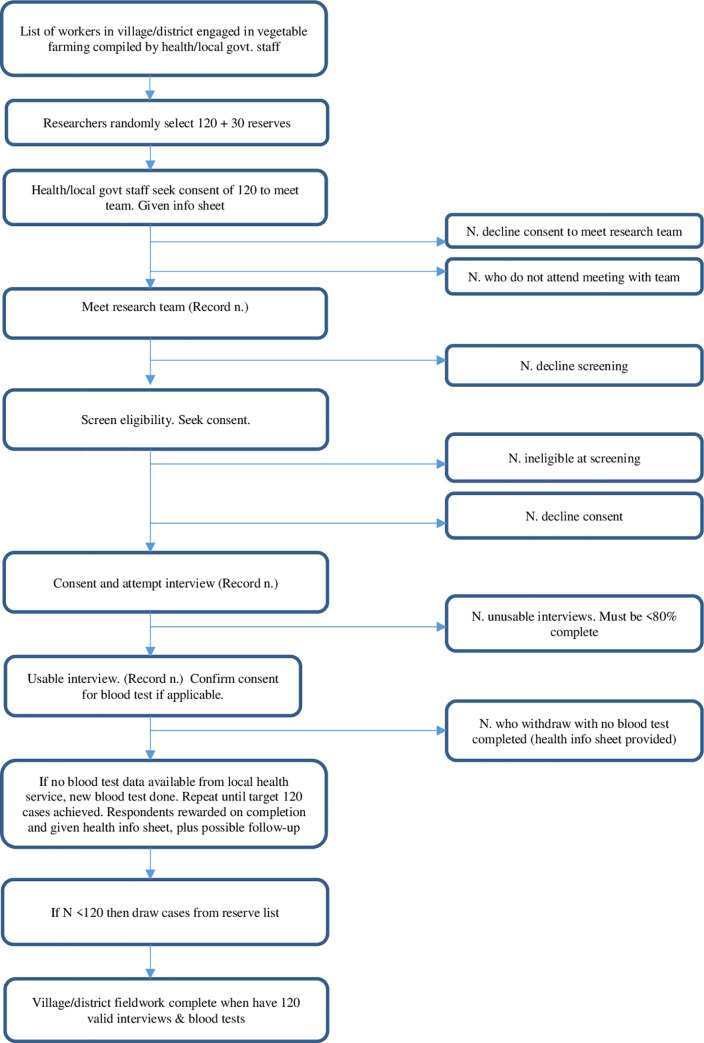
Flow diagram of selection and fieldwork at village/district level (3 districts per country).

A flow diagram illustrating the fieldwork process is shown in Fig 1.

### Research aim

To investigate the potential health impacts of pesticides use on agricultural workers’ health.

### Outcomes

1. a. Acetyl cholinesterase concentrations
  b. Self-reported ill-health measures separately and together

### Predictors

2. Practices/Behaviours
  - Likely to increase exposure
  - Likely to decrease exposure
3. Knowledge

### Covariates

1. Location:
  country, district, type of area, farm type, crop
2. Personal characteristics
  Age, gender, income, socio-economic status (SES), migration status, employment status, education, ability to read and write, distance between home and farm, hours worked.

### Analysis outline

Data will be summarised using appropriate descriptive statistics; we will report on associations between agricultural methods, health knowledge, health-protective practices, self-reported illness and blood test results.

Data from each country will be analysed separately before considering whether an analysis of pooled data is appropriate.

1. Description of all variables: frequencies, or ranges and measures of central tendency
2. Combining structured interview responses to give overall measures of: outcomes, knowledge, practices/behaviours and exposures. The variables to be combined will be listed *a priori*.
  Measures of ill-health, knowledge, behaviours and exposures will be described separately and together.
3. Bivariate explorations will be used to examine the variables shown in Table 1.

**Table 1:** Contingency tables for each comparison.

	Ill-health measures	Exposure	Practices/behaviours	Knowledge	Cholinesterase concentration
Ill-health measures					
Exposure					
Practices/behaviour					
Knowledge					
Cholinesterase concentration

4. Multivariate analyses
  Primary outcomes: AChE concentrations, ill-health measures, separately & together
  Covariates to be tested one by one
  Covariates with P<0.20 will be entered into exploratory models

a) Logistic regression: high AChE concentration, any ill-health, each health issue (modelling strategy to be based on backwards LR, -2LL, VIF, check enter model)

Predictors:

1. Behaviours
  a. Likely to increase exposure
  b. Likely to decrease exposure
2. Knowledge score
3. Cholinesterase concentrations (when exploring ill-health)
4. Significant covariates from one-by-one testing will include: location, SES, education, literacy (ability to write), age, gender, hours worked, distance between home and farm.

Sensitivity analyses will, in turn, exclude:

Migrant workers
Earlier diagnosis of pesticide poisoning

A priori sub-group analyses:

1. Each country
2. Self-employed
3. Lowest SES or income category
4. No formal education

b) Meta-analysis of all countries. A random-effects meta-analysis approach incorporates an assumption that the different studies are estimating different, yet related, intervention effects. It is assumed that there is a similarity in the patterns found in, for example, a study of pesticides impacts in three countries, but that the effects found will not be identical due to contextual factors. [65]. The approach provides a way to estimate how far differences reflect true effects in data from multiple countries, rather than chance differences arising from sampling, Meta-analysis of continuous variables with data that are not normally distributed will not be undertaken. We anticipate equal numbers of responses from each country, reducing the effects of study weighting by size. If heterogeneity of country findings is moderate or low, we shall consider multilevel modelling of pooled data to account for country in addition to location, and farm and crop type. Heterogeneity will be assessed according to the GRADE guidelines, including: variation in estimates across the different countries, particularly where confidence intervals do not overlap, and the *I*^*2*^ test of heterogeneity. We shall take 50–90% as substantial, and 75–100% *I*^*2*^ values as considerable heterogeneity [66]. Any *I*^*2*^ values over 90% will jeopardise the value and reporting of a meta-analysis.

*A posteriori* subgroup analyses will be used to explore links for future studies, rather than study outcomes. It is difficult to speculate on the nature of such analyses, but they might include exploration of the data by respondents’ ages or gender.

## Discussion

### Benefits of the study

The study will provide baseline knowledge on health impacts of pesticides use, as well as farm workers’ agricultural practices, health knowledge, health-protective behaviours and self-reported illnesses that will assist policy makers and healthcare planners in the three countries. The findings should help inform recommendations for health improvement, especially in the areas of workers’ self-care and safe work practices. Measuring the magnitude of the problem of pesticide exposure (as cholinesterase concentrations) in agricultural workers should provide valuable information on targeting and prioritisation for health workers responsible for local pesticides monitoring programmes. An assessment of similarities and differences between the three countries will be helpful in identifying best practice, and improving regulatory arrangements and monitoring systems.

More generally, this initial project will help develop cooperation and research capacity in the trans-national research network. In time it is hoped that the network can be developed further, so that funding can be obtained for follow-on projects on pesticides use and other health-related topics.

### Limitations

Because this study is a development project, it must be conducted with modest resources and a tight time schedule. This limits the scale and generalisability of the study. Other operational considerations include the need to proceed against the background of the COVID-19 pandemic, so that we must restrict data collection to accessible districts in a single province in each country. This means that we cannot use probability sampling for all levels of site and respondent selection, and affects the levels of statistical significance assumed in our analysis. As with all non-randomised studies, we cannot assume that association equates to causation. Rather, our aim is to identify behaviours predictive of adverse outcomes, and suggest strategies to target these behaviours.

### Risks

In the ongoing COVID-19 epidemic, there is a risk that recruitment targets will not be met. The safety of researchers is paramount, and it may be that data will have to be collected from villages and districts in close proximity to universities. This may render our sample unrepresentative of the national populations.

### Timeline

In accordance with the conditions of GCRF funding, expenditure for the fieldwork phase of this project needs to be incurred by October 15^th^ 2021. Data analysis and writing of a report and papers can continue later into 2021. Because the individual country teams are at different stages of the research process, it is not possible to specify a definitive timetable for all. However, in general terms the following will apply.

- The structured interview instrument to be finalised by end of March 2021
- All REC applications to be submitted by end May 2021
- Collection of main background information and an outline review of relevant literature should be completed by end of May 2021.
- Study sites should have been identified and any necessary permission from local government officials or others to proceed obtained by end of July 2021.
- Fieldwork (interviews and blood tests) should have been completed by mid-October 2021.

At the time of writing, options for follow-on funding are being curtailed by the COVID-19 crisis. One consequence of the UK Government’s decision to reduce its overseas development aid budget from 0.7% to 0.5% of GDP is that many Global Challenge Research Fund calls have been suspended, and no funds will be allocated for internal disbursement by UK universities in 2021–22. The research team will endeavour to find other funding sources to take our network forward.
